# Overexpression of *salt-induced protein* (*salT*) delays leaf senescence in rice

**DOI:** 10.1590/1678-4685-GMB-2017-0365

**Published:** 2019-01-31

**Authors:** Keming Zhu, Huimin Tao, Shuo Xu, Kaixia Li, Sundus Zafar, Wei Cao, Yanhua Yang

**Affiliations:** 1 Jiangsu University Jiangsu University Institute of Life Sciences ZhenjiangJiangsu People’s Republic of China Institute of Life Sciences, Jiangsu University, Zhenjiang, Jiangsu, People’s Republic of China

**Keywords:** Leaf senescence, *salt-induced protein* (*salT*), rice, stay-green

## Abstract

Senescence, a highly programmed process, largely determines yield and quality of crops. However, knowledge about the onset and progression of leaf senescence in crop plants is still limited. Here, we report that salt-induced protein (*salT*), a new gene, may be involved in leaf senescence. Overexpressing *salT* could prolong the duration of leaves with higher concentrations of chlorophyll compared with the wild type. Moreover, overexpression of *salT* could delay the senescence of rice leaves though the inhibition of senescence associated genes (SAGs). Overall, the characterization of *salT* suggested that it is a new gene affecting the leaf senescence induced by natural and dark conditions.

## Introduction

Senescence is the final phase of plant development, in which the plant goes through a series of programmed cell death (PCD) processes (Cao *et al.*, 2003){Cao, 2003 #1}. This process combines chlorophyll degradation with carotenoid retention or anthocyanin accumulation (Park *et al.*, 2007) so the leaves of plant generally change from green to yellow during senescence. It has been noted for long that leaf senescence is a major determinant of yield for many crops (Richards, 2000; Long *et al.*, 2006; Yang and Zhang 2006). Delaying leaf senescence has usually been considered to be associated with the retention of the high photosynthetic capacity and yield increment (Thomas and Howarth, 2000; Masclaux-Daubresse and Chardon, 2011; Wu *et al.*, 2016).

Rice (*Oryza sativa* L.) is one of the most important crops in the world and provides nearly half of the calories consumed by humankind (Zuo and Li, 2014). The leaf is the most important source organ for rice, with 60-80% of the nutrients required for grain filling after heading provided by leaf photosynthesis (Lu *et al.*, 1988). Early-senescence of leaves will seriously reduce rice yield, disrupt the filling dynamics, and reduce grain quality (Zhao *et al.*, 2014). The production can be increased by 2% with delaying rice senescence by one day (Liang *et al.*, 2014). Therefore, finding an economic and effective method to delay leaf senescence in rice production can greatly improve the yield of rice.

During leaf senescence, the expression of senescence associated genes (SAGs) is up-regulated, which is a hallmark of leaf senescence (Kajimura *et al.*, 2010). More than a thousand SAGs have been isolated, including transcription factors, signal transduction components, proteases, metabolic enzymes and various transporters of nutrients (Watanabe and Imaseki, 1982; John *et al.*, 1997; Lee *et al.*, 2001; Buchanan-Wollaston *et al.*, 2003; Gregersen and Holm, 2007, Liu *et al.*, 2008; Hayashi *et al.*, 2015; Wu *et al.*, 2016). Some important genes have been cloned and functionally characterized. *STAY-GREEN* (*SGR*) is an important member of the metabolic pathway of chlorophyll degradation, which encodes a chloroplast transit peptide and regulates chlorophyll degradation by inducing light-harvesting proteins of photosystem II (LHCPII) disassembly through direct interaction (Jiang *et al.*, 2007; Hörtensteiner; 2009). *RED CHLOROPHYLL CATABOLITE REDUCTASE 1* (*RCCR1*) encodes a red chlorophyll catabolite reductase, plays a key role in the chlorophyll degradation pathway, and strongly participates in senescence (Tang *et al.*, 2011). *NON-YELLOW COLORING 1* (*NYC1*) encodes the chlorophyll b oxidation-reduction enzyme, and chlorophyll b cannot be degraded in the *nyc1* mutant because of abnormal binding of light-harvesting chlorophyll and carotene (Kusaba *et al.*, 2007). *NON-YELLOW COLORING 3* (*NYC3*) encodes a plastid-targeted α/β folding proteolytic enzyme that affects chloroplast structure, and the *nyc3* mutant exhibits a senescence phenotype (Morita *et al.*, 2009). *OsNAP* encodes a transcription factor with a crucial role in regulating the senescence process, as demonstrated by the delayed senescence phenotype of *Osnap* mutants (Liang *et al.*, 2014). In general, the onset and progression of leaf senescence is influenced by a number of endogenous and external factors. The mechanism of the onset of leaf senescence, especially the mechanism of delayed senescence in rice, is still largely unknown.

Here, we investigated the role of the *salt-induced protein* (*salT*) gene in rice. In our previous studies, we found OsISP specifically expressed in *indica* rice and the protein was identified as a salt-induced protein (salT) (Zhu *et al.*, 2014). However, salT function is still not clear in rice. Here, we found that overexpression of *salT* in rice could delay the senescence of leaf by inhibiting the expression of SAGs and CDGs.

## Material and Methods

### Plant materials and growth conditions

All rice lines used in the study were derived from the *japonica* cultivar Nipponbare. Rice plants were cultivated in the experiment field at Jiangsu University, Zhenjiang, Jiangsu, during the natural growing season except where specifically indicated. For hydroponic culture, rice seedlings were grown in a constant-temperature incubator with light/dark of 16/8 h and 30-28 °C, with approximately 200 μmol photons/m^2^/s photon-density and 70% humidity. For dark incubation, the third fully expanded leaves of each genotype were excised and incubated under continuous darkness at 28 °C. All experiments were carried out using the same location of the leaves. The samples were collected with a sharp scalpel to minimize the impact of wounding, immediately frozen in liquid nitrogen, and stored at -72 °C until needed for RNA isolation and chlorophyll extraction. All experiments were repeated three times independently.

### Vector construction and plant transformation

Total RNA from rice leaf tissues (Zhonghua11) was extracted using TRIzol reagent (Invitrogen, Carlsbad, CA, USA). After removal of genomic DNA contamination by DNase I (TaKaRa, Dalian, China), 200?ng of poly(A)+ mRNA was converted into cDNA by MMLV Reverse Transcriptase (Vazyme, Nanjin, China). The cDNA template was subsequently used for PCR analysis. For *salT* (Os01g0348900), a full-length cDNA was obtained using the primers 5’-ATGACGCTGGTGAAGATTGG-3’ and 5’-TCAAGGGTGGACGTAGATGC-3’.

To construct a vector for the constitutive expression of *salT*, a 438 bp full-length *salT* cDNA was PCR amplified from its cDNA clone with the primers 5’-AAGTCGACATGACGCTGGTGAAGATTGG-3’ and 5’-AACTGCAGTCAAGGGTGGACGTAGATGC-3’, and inserted into the *Sal* I/ *Pst* I site of pCAMBIA1300- Actin1-ocs, creating an overexpression vector, *salT-OE*. The inserted sequences were confirmed by restriction enzyme digestion and sequencing. To construct the *salT* RNAi vector, part of the *salT* cDNA was PCR amplified from its cDNA clone with the primers 5’-AAGGATCCATGACGCTGGT GAAGATTGG-3’ and 5’-AAGTCGACATGGGTTCCAG AAATCTCCTT-3’ and inserted into the *Bam*H I/*Sal* I and *Xho* I /*Bgl* II sites of the pUCCRNAi vector. Then, the pUCCRNAi vector was cut with *Pst* I and inserted into the *Pst* I site of pCAMBIA1300-Actin1-ocs, creating a *salT* RNAi vector, *salT-*RNAi. The inserted sequences were confirmed by restriction enzyme digestion and sequencing. The two binary plasmids were introduced into *Agrobacterium* tumefaciens EHA105 by electroporation and transformed into rice according to a published method (Hiei *et al.*, 1994; Jeon *et al.*, 2000).

### DNA, RNA extraction, and qRT-PCR

Genomic DNA was extracted from rice leaves using the CTAB method and total RNA was extracted using the TRIzol reagent (Invitrogen). RNA was reversely transcribed from 3 μg of total RNA with the M-MLV reverse transcriptase (Vazyme) according to the manufacturer’s instructions. The qRT-PCR asays were carried out in a total volume of 20 μL, each containing 2 μL of cDNA (200 ng), 10 μL of SYBR Green Master Mix (Vazyme), 0.4 μL of 50ROX Reference Dye I, 0.4 μL of primers (10 μM) and 7.2 μL of H_2_O. Cycling conditions included a hot start (5 min at 95 °C), followed by 40 cycles of 95 °C for 10 s, and 60 °C for 30 s, and finished with 95 °C for 15 s, 60 °C for 60 s, and 95 °C for 15 s. Amplification was performed using an ABI7300 PCR thermocycler (Applied Biosystems, USA). Rice *Actin1* was chosen as a control to normalize all data. For analysis, a threshold was set for the change in fluorescence at a point in the linear PCR amplification phase. Melt curve analysis was performed to ensure a single product species. All experiments were done in duplicate for both target gene and internal control and were repeated three times independently. For the CT calculation to be valid, the amplification efficiencies of the target and reference must be approximately equal. The relative expression levels of genes were calculated using the 2^-Ä^ΔCt method (He *et al.*, 2016; Tan *et al.*, 2015). The statistical significance was analyzed by Student’s *t*-test. The primer sequences that were used are listed in Table S1. PCR products were separated by electrophoresis on 1.0% (w/v) agarose gels.

### Chlorophyll measurement

Fresh rice leaves (20 mg) were chilled with liquid nitrogen and ground to a powder. Chlorophyll was extracted with 80% acetone until the residue turned to white color, and diluted to a final volume of 25 mL. Chlorophyll was determined by measuring absorbance at 663 and 645 nm using a W14546 spectrophotometer (Xin Mao Instrument, Shanghai, China). The chlorophyll content was calculated according to the formula (Ca = 12.7 OD663 - 2.69 OD645, Cb = 22.9 OD645 - 4.68 OD663, Ct = 8.02 OD663 + 20.21 OD645). Three seedlings of each sample were used in the chlorophyll assay. Statistical significance was analyzed by Student’s *t*-test. Values are reported as mean ± standard deviation of 10 measurements.

## Results

### Overexpressing *salT* delayed the senescence of rice leaves

**Figure 1:**
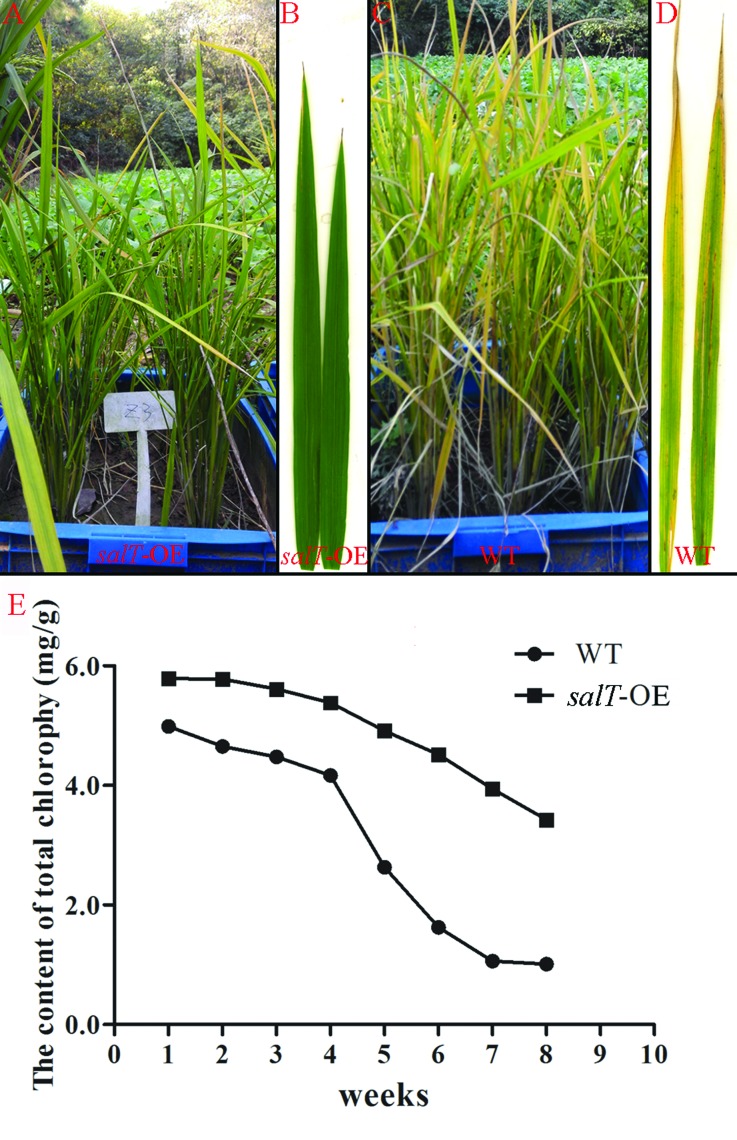
Phenotype of wild-type and T_0_ transgenic plants carrying *salT* overexpression constructs (*salT*-OE) in the mature stage. (A) Phenotypes of the T_0_ *salT*-OE plants in the mature stage. (B) Flag leaf of the *salT*-OE plants keeps green in the mature stage. (C) Phenotypes of the wild type in the mature stage. (D) Flag leaf of the wild type turned yellow in the mature stage. (E) Chlorophyll contents of leaves of age-matched wild-type and *salT*-OE plants.

The OsISP protein was a protein marker for *indica* rice varieties, and was identified as salt-induced protein (salT), however the function of salT is still not clear in rice (Zhu *et al.*, 2014). To further investigate its function, we constructed *salT*-overexpression (*salT*-OE) and *salT*-RNAi vectors, and generated transgenic plants by *Agrobacterium* mediated transformation (Hiei *et al.*, 1994; Jeon *et al.*, 2000). A total of eight positive *salT*-OE transgenic plants and 16 positive *salT*-RNAi transgenic plants were obtained. All eight *salT*-OE transgenic plants (T_0_) maintained a stay-green phenotype in the mature stage (Figure 1A,B), but the phenotype of *salT*-RNAi transgenic rice was similar to the wild type (data not shown). Thus, we used the *salT*-OE transgenic rice lines for our study.

**Figure 2:**
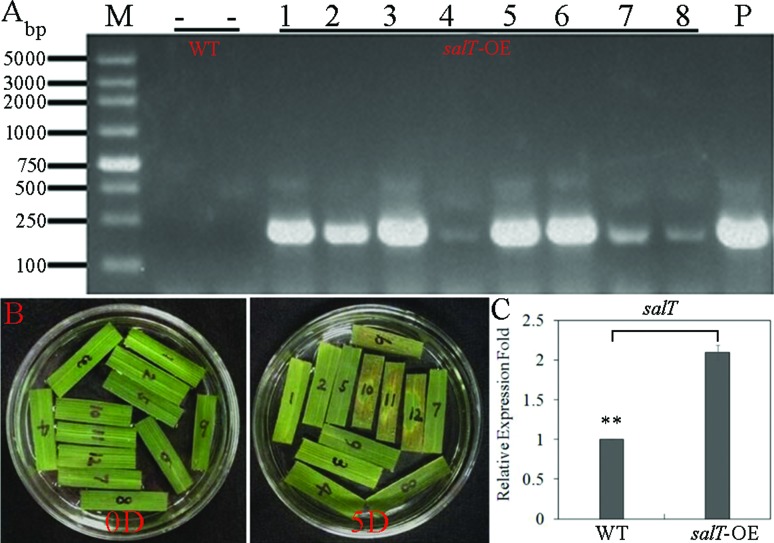
Analysis of the *salT*-OE transgenic plants. (A) PCR analysis of *hyg* gene in the T_0_ *salT*-OE transgenic plants. M: DL2000 plus DNA maker; -: untransformed wild-type plants; 1-8: T_0_ *salT*-OE transgenic plants; P: pCAMBIA1300-Actin1-ocs plasmid. (B) Leaf assay of hygromycin resistance in transgenic rice plants. 1-8: T_0_ *salT*-OE transgenic plants; 9-12: untransformed wild type plants. (C) Transcript levels analysis the expression of *salT* gene between wild type and *salT*-OE transgenic plant. *p < 0.05,*p < 0.01,*p < 0.001. Student’s *t*-test was used to generate *p-*value.

Total DNA was extracted from T_0_ transgenic rice plant and wild type leaves. In all T_0_ transgenic rice plants a 220 bp fragment could be identified with hygromycin (*hpt*) gene-specific primers, which was similar to the positive control. However, no PCR products were detected in the wild type plants (Figure 2A). Moreover, we tested hygromycin resistance in excised leaves with hygromycin detection solution. All excised leaves from *salT*-OE transgenic plants stayed green and showed resistance to hygromycin while the leaves from untransformed control plants turned brown and died, indicating no resistance to hygromycin (Figure 2B). Furthermore, we extracted RNA from a *salT*-OE transgenic plant, and the qRT-PCR results showed that the *salT* gene expression in *salT*-OE transgenic plant was about two times higher than that in wild type (Figure 2C). These results indicate that we successfully constructed *salT*-OE transgenic plants.

Phenotypic analysis of *salT*-OE transgenic plants was carried out. As shown in Figure 1, at the mature stage of rice, the wild type leaves turned yellow (Figure 1C and D) while the leaves of *salT*-OE rice had a stay-green phenotype (Figure 1A,B). We also quantified the chlorophyll content of flag leaves from the first week to the eighth week of the filling stage. During this period, the chlorophyll content of the leaves decreased gradually, but in *salT*-OE rice it decreased much slower than in wild type (Figure 1E). These results showed that overexpression of the *salT* gene in rice could delay the senescence of leaves.

### *salT* inhibits the expression of senescence associated genes

**Figure 3:**
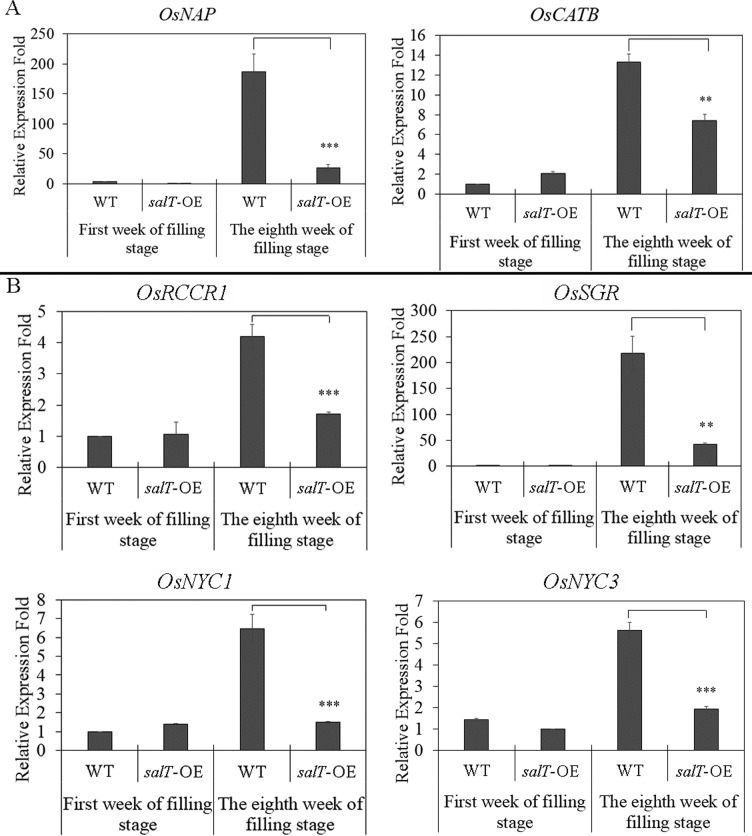
Overexpression of *salT* inhibits the expression of senescence genes. (A) Relative transcript levels of senescence-associated genes in wild type and *salT*-OE plants during the filling stage. (B) Relative transcript levels of chlorophyll degradation-related genes (*OsRCCR1*, *OsSGR*, *OsNYC1* and *OsNYC3*) of age-matched wild type leaves and *salT*-OE plants. Overexpression of *salT* gene delays dark-induced leaf senescence. Transcript levels are expressed relative to rice *Actin1* in each sample, and values are reported as mean ± SD (n = 3). *p < 0.05,*p < 0.01,*p < 0.001. Student’s *t*-test was used to generate *p*-value.

The expression of SAGs are up-regulated during leaf senescence (Kajimura *et al.*, 2010). Thus, we used quantitative real-time PCR to detect two SAGs, *OsNAP* and *OsCATB*, in the flag leaves from wild type and *salT*-OE rice during the filling stage. As shown in Figure 3A, during the first week of the filling stage, the two genes were barely expressed in flag leaves. In the eighth week of the filling stage, the expression level of these two genes increased rapidly in the wild type, but not in the *salT*-OE plants. This result indicated that *salT* could inhibit leaf senescence by inhibiting the expression of SAGs.

Chlorophyll plays a central role in photosynthesis, and its degradation is an important phenomenon in leaf senescence (Morita *et al.*, 2009). Therefore, we also quantified the expression levels of four chlorophyll degradation-related genes (CDGs), *OsSGR*, *OsNYC1*, *OsNYC3*, and *OsRCCR1*, by real-time PCR during the filling stage. Our results showed that the expression of the four genes had almost no difference between wild type and *salT*-OE plants in the first week of the filling stage. At the eighth week of the filling stage, the expression level of these genes increased sharply in wild type. In *salT*-OE plants, the expression levels of the four genes also increased, but they were lower than in wild type (Figure 3B). This results suggests that overexpression of *salT* could keep leaves green through inhibiting chlorophyll degradation.

### Responses of *salT* to the dark treatment

Dark treatment is the simplest and most efficient method to induce leaf senescence. While the detached leaves of Nipponbare (wild type) turned yellow 3 days after dark incubation (DAD), *salT*-OE retained the greenness at 5 DAD (Figure 4A). The chlorophyll content of rice seeding was examined during dark incubation. Initial chlorophyll contents were similar between wild type and *salT*-OE, but a prominent decrease was observed at 7 DAD in wild type (Figure 4B). The qRT-PCR results showed that two CDGs, *OsSGR*, and *OsRCCR,* had lower expression in *salT*-OE than in wild type plants after 7 DAD (Figure 4C). These observations indicate that *salT*-OE plants retain more chlorophyll than the wild type during senescence through inhibiting the expression of CDGs.

**>Figure 4:**
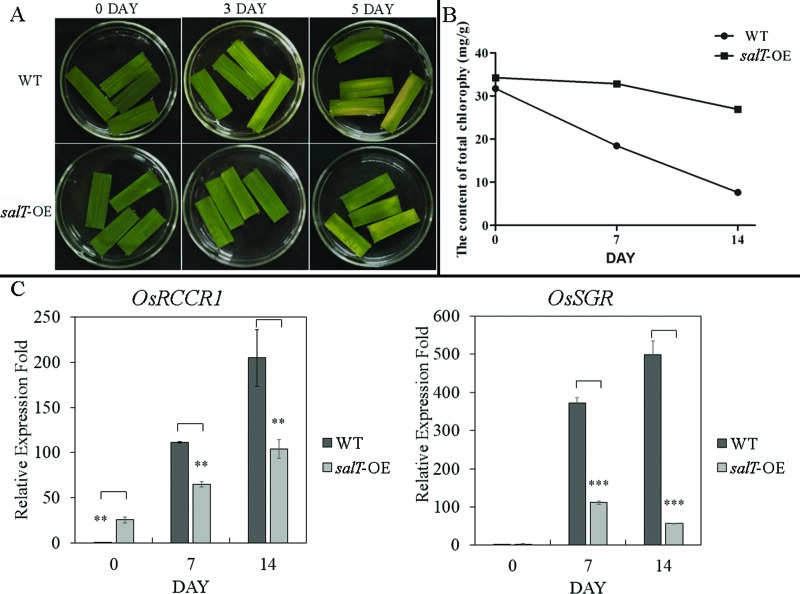
Changes in the chlorophyll content of wild type leaves and of *salT*-OE lines during dark-induced senescence. (A) Changes in leaf color in detached leaves stored in darkness. (B) Chlorophyll contents and (C) relative transcript levels of chlorophyll degradation-related genes (*OsSGR* and *OsRCCR1*) of wild-type and *salT*-OE plants cultivated in darkness. Transcript levels are expressed relative to rice *Actin1* in each sample, and values are reported as mean ± SD (n = 3). *p < 0.05,*p < 0.01,*p < 0.001. Student’s t-test was used to generate *p-*value.

## Discussion

Leaf senescence, the final stage of leaf development, occurs autonomously in an age-dependent manner and leads to the death of a cell or an organ. The aging process that leads to senescence and limitation of life is a common biological phenomenon in most organisms (Woo *et al.*, 2001). Up to now, it is clear that leaf senescence is controlled by genes, and many leaf senescence genes in Arabidopsis, rice, maize, and barley have been cloned (Schippers, 2015; Raines *et al.*, 2016; Sakuraba *et al.*, 2016; Deng *et al.*, 2017). However, there are few studies on delayed leaf senescence, especially in rice (Ren *et al.*, 2007; Hörtensteiner, 2009; Morita *et al.*, 2009). In this study, we found that *salT* could slow down the degradation of chlorophyll by reducing the expression of SAGs and CDGs, which could delay the senescence of leaves (Figures 1 and 2).

During leaf senescence, the most striking phenotypic change is the yellowing of the leaf caused by the preferential breakdown of chlorophyll and chloroplasts (Hilditch *et al.*, 1989; Woo *et al.*, 2001). The change in leaf color and chlorophyll content is integrally related to leaf senescence and is widely used in the quantification of senescence. CDRGs (*OsSGR*, *OsNYC1*, *OsNYC3*, and *OsRCCR1*) play important roles in regulating chlorophyll degradation, and the transcription levels of these genes are up-regulated during natural and dark-induced leaf senescence (Jiang *et al.*, 2007; Park *et al.*, 2007; Sato *et al.*, 2009; Tang *et al.*, 2011; Rong *et al.*, 2013; Liu *et al.*, 2016). The *sgr* mutant maintains greenness during rice leaf senescence, while overexpressing *SGR* in rice produces yellowish-brown leaves (Park *et al.*, 2007). The *SGR* gene encodes a chloroplast protein and is essential for the initiation of chlorophyll breakdown in plants (Park *et al.*, 2007; Hörtensteiner, 2009; Liu *et al.*, 2016).

The rice leaf *nyc1* mutant also stays green during senescence, because chlorophyll degradation is impaired in the *nyc1* mutant (Kusaba *et al.*, 2007), and NYC1 is suggested to play essential roles in the regulation of LHCII and thylakoid membrane degradation during senescence (Kusaba *et al.*, 2007). *OsNYC3* encodes a plastid-targeted α/β hydrolase-fold family protein with an esterase/lipase motif, which affects chloroplast structure (Morita *et al.*, 2009). *OsRCCR1* encodes a red chlorophyll catabolite reductase, which plays a key role in the chlorophyll degradation pathway. The transcription level of *OsRCCR1* is much lower in young leaves, but is about 20-fold higher in senescent leaves (Tang *et al.*, 2011). These evidences suggest that *OsSGR*, *OsNYC1*, *OsNYC3*, and *OsRCCR1* play important roles in regulating chlorophyll degradation during leaf senescence.

In this study, we found that the chlorophyll degradation rate of *salT*-OE plant leaves was lower than that of wild type during the mature period of rice (Figure 1E). Our results sbowed that the transcription levels of *OsSGR*, *OsNYC1*, *OsNYC3*, and *OsRCCR1* were down-regulated during rice leaf senescence (Figure 3B). In addition, disadvantageous environmental factors, such as darkness, can also trigger senescence during leaf development (Zhang and Zhou, 2013). The results in Figure 4A showed that darkness can induce senescence in wild type, while overexpression of *salT* could delay leaf senescence in detached leaves. Moreover, the chlorophyll degradation rate of *salT*-OE plant leaves is lower than that of wild type during dark incubation (Figure 4B). The expression of *OsSGR* and *OsRCCR1* was also inhibited with darkness treatment (Figure 4C). Our results showed that the leaves of *salT*-OE plants can stay green, because overexpression of the *salT* gene could inhibit the expression of CDGs (*OsSGR*, *OsNYC1*, *OsNYC3*, and *OsRCCR1*), which inhibits chlorophyll degradation during senescence in rice.

Previous studies showed that some genes, such as SAGs, are up-regulated in senescent leaves. For example, the expression of *AtNAP* increases with aging; overexpression of *AtNAP* in wild type triggers precocious senescence and significantly blocks the function of the transcription factor that delays senescence (Guo and Gan, 2006). *OsCATB* is significantly induced by ABA (Abscicic Acid) (Agrawal *et al.*, 2001; Ye *et al.*, 2011). As a matter of fact, the up-regulation of SAGs is one of the hallmarks of leaf senescence (Kajimura *et al.*, 2010). The results in Figure 3A show that the expression of SAGs (*OsNAP* and *OsCATB*) was not different from the wild type before leaf senescence, but was down-regulated during rice leaf senescence. This result indicates that the overexpression of *salT* could inhibit the expression of SAG gene and inhibit leaf senescence.

In general, overexpression of the *salT* gene could delay leaf senescence in rice by inhibiting the expression of SAGs and CDGs. Thus, *salT* is a new anti-aging factor gene, but its mechanism is still unclear and needs to be further explored.

## Supplementary material

The following online material is available for this article:

Table S1 Primers used in this study.
