# Supplementary material for: Overexpression of salt-induced protein (salT) delays leaf senescence in rice
Source: Genet Mol Biol. 2019 Jan 31;42(1):80–6. doi: 10.1590/1678-4685-GMB-2017-0365 (PMC6428123; doi:10.1590/1678-4685-GMB-2017-0365)
Supplement: Supplementary file 1 [file 1415-4757-GMB-1678-4685-GMB-2017-0365-s001.pdf]

Supplementary material to "Overexpression of *salt-induced protein* (*salT*) delays leaf senescence in rice"

Table S1 - Primers used in this study

Gene	F/R	Primer sequence (5′-3′)
Actin1	F	CTCGTCTGCGATAATGGAACTG
	R	CTCGTTGTAGAAGGTGTGATGCC
salTQ	F	GACATCAGTGTGCCACCCAA
	R	CAGAGGAGCCCAATTTAATCTCT
OsSGR	F	AGGGGTGGTACAACAAGCTG
	R	GCTCCTTGCGGAAGATGTAG
OsRCCR1	F	CGCATTTCCTCATGGAATTT
	R	CTTCTCACGCTGTTTGTCCA
OsNYC1	F	AGGAGGGCTTGTCAGTTGCG
	R	ACCTTTGTTTGTGCCAGCGTTA
OsNYC3	F	TGTCGTTGCCATGTGAAGAT
	R	TTGGTCACGCCACAAATCTA
OsNAP	F	CCCAAGGGCACCAAGACCAACT
	R	AGCACCCAGTCATCCAGCCTCA
OsCATB	F	GGTGGGTTGATGCTCTCTCA
	R	ATTCCTCCTGGCCGATCTAC
hpt	F	TCGTTATGTTTATCGGCACTTTG
	R	GAACCCGCTCGTCTGGCTAA
salT-OE	F	AAGTCGACATGACGCTGGTGAAGATTGG (*Sal* I)
	R	AACTGCAGTCAAGGGTGGACGTAGATGC (*Pst* I)
salT-RNAi	F	AAGGATCCATGACGCTGGTGAAGATTGG (*Bam*H I)
	R	AAGTCGACATGGGTTCCAGAAATCTCCTT (*Sal* I)
